# Protein Nano-Object Integrator (ProNOI) for generating atomic style objects for molecular modeling

**DOI:** 10.1186/1472-6807-12-31

**Published:** 2012-12-05

**Authors:** Nicholas Smith, Brandon Campbell, Lin Li, Chuan Li, Emil Alexov

**Affiliations:** 1 Computational Biophysics and Bioinformatics, Department of Physics, Clemson University, Clemson, SC, 29634, USA

**Keywords:** Biological macromolecules, Electrostatic calculations, Molecular modeling, Nano technology, DelPhi, Poisson-Boltzmann equation

## Abstract

**Background:**

With the progress of nanotechnology, one frequently has to model biological macromolecules simultaneously with nano-objects. However, the atomic structures of the nano objects are typically not available or they are solid state entities. Because of that, the researchers have to investigate such nano systems by generating models of the nano objects in a manner that the existing software be able to carry the simulations. In addition, it should allow generating composite objects with complex shape by combining basic geometrical figures and embedding biological macromolecules within the system.

**Results:**

Here we report the *Protein Nano-Object Integrator (ProNOI)* which allows for generating atomic-style geometrical objects with user desired shape and dimensions. Unlimited number of objects can be created and combined with biological macromolecules in Protein Data Bank (PDB) format file. Once the objects are generated, the users can use sliders to manipulate their shape, dimension and absolute position. In addition, the software offers the option to charge the objects with either specified surface or volumetric charge density and to model them with user-desired dielectric constants. According to the user preference, the biological macromolecule atoms can be assigned charges and radii according to four different force fields: Amber, Charmm, OPLS and PARSE. The biological macromolecules and the atomic-style objects are exported as a position, charge and radius (PQR) file, or if a default dielectric constant distribution is not selected, it is exported as a position, charge, radius and epsilon (PQRE) file. As illustration of the capabilities of the *ProNOI*, we created a composite object in a shape of a robot, aptly named the Clemson Robot, whose parts are charged with various volumetric charge densities and holds the barnase-barstar protein complex in its hand.

**Conclusions:**

The *Protein Nano-Object Integrator (ProNOI)* is a convenient tool for generating atomic-style nano shapes in conjunction with biological macromolecule(s). Charges and radii on the macromolecule atoms and the atoms in the shapes are assigned according to the user’s preferences allowing various scenarios of modeling. The default output file is in PQR (PQRE) format which is readable by almost any software available in biophysical field. It can be downloaded from: http://compbio.clemson.edu/downloadDir/ProNO_integrator.tar.gz

## Background

The enormous progress made in experimental techniques for 3D structural determination of biological macromolecules and their assemblages resulted in quick expansion of the Protein Data Bank (PDB), which currently contains more than 83,000 entries [1,2]. Combined with the ever increasing accuracy of comparative and *ab-initio* modeling, nowadays the biophysical community has access to huge amount of structural data. In a long run, it is anticipated that the entire structural universe (experimentally determined structures and high quality models) of human genome and other selected organisms will be available [3-5]. This structural information is crucial for understanding macromolecular function, details of the biochemical reaction, electron and proton transfer phenomena and many other biological processes occurring in the cell.

Given the 3D atomic structure of a macromolecule, various computational approaches can be applied to model the above mentioned processes. Perhaps the most popular is molecular dynamics (MD) simulation, which takes the atomic structure as an input and applies computational protocol to simulate its time-dependence using particular force field [6-8]. Other approaches use static structures (or pre-generated ensemble of structures) to calculate the electrostatic potential distribution and to calculate electrostatic energies [9-18]. The 3D structures are used to predict pKa’s of ionizable groups [19-21], to calculate the conformational energy [22], to model salt dependence of protein stability and binding [23-26], and to infer the proton pathway [27]. The knowledge of the atomic structure of a macromolecule is crucial for correct predictions of the effect of mutations on protein stability and affinity [28,29]. With the progress made in genome sequencing and detection of missense mutations in sick individuals, the 3D structure of the protein carrying the disease-causing defect is a very important component for understanding the molecular mechanism of the disease and for seeking for a possible treatment [30-32].

At the same time, with the progress and development of nanotechnology, researchers frequently have to model biological macromolecules in conjunction with nano-objects. Such mixed/hybrid systems occur in medicine where researchers need to understand the interaction between biological macromolecules and implants, the implants being made of metal or other solid state materials [33-35]. The shape of these implants varies from such simple shapes as a plate to very complex shapes [36]. The properties of these objects vary as well spanning from pure conductor (metal) [37,38] to an insulator (plastic) [39,40]. Biochemists frequently investigate the properties and characteristics of biological macromolecules via experimental devices or techniques involving nano-objects. Typical examples are experiments involving atomic force microscope (AFM), where the tip of the microscope, in a shape of cone, is used to probe the molecular surface [41-43]. Other examples include immobilization of biological macromolecules on various surfaces for either binding affinity or conformational changes investigations [44,45], for protein microarrays [46], or for drug-affinity chromatography [47].

However, the atomic structures of the above-mentioned nano-objects are typically not available in an acceptable format, or the modular size and shape of such objects prevents the creation of usable standard models. An attempt was made in the DelPhi distribution (version 4 and higher) to allow for modeling of four basic types of geometrical figures namely sphere, cylinder, cone and parallelepiped. However, no visualization and manipulation was allowed and DelPhi objects were not transferrable to other software available in the computational community. To overcome these limitations, these hybrid nano-systems need to be rendered in a widely acceptable format that can be used by existing simulation software. Here we report such stand-alone software enriched with GUI based on Jmol. The software, the *Protein Nano-Object Integrator (ProNOI)* allows the users to create atomic-style geometrical objects and to integrate them with standard biological macromolecules. The atomic-style presentation offers huge advantage because, in principle, these systems can be outputted to any existing software that uses PDB files. This enables the objects’ properties to be adjusted according to the user requirements in order to model the electrostatic and mechanical characteristics of the hybrid structure using the appropriate force field parameters.

## Implementation

The main body of the GUI was designed using an interface coded in Java which communicates with a C++ command line program in the background to generate the atomic-style objects. The program uses the Java Swing libraries for the visual interface design and encapsulates the BioJava implementation of the Jmol molecular viewer [48] in order to provide the user with a clear visual representation of their protein(s) and associated nano-objects.

Once the program boots, it allows the user to either insert objects into an entirely empty file or open up their own PDB/PQR file for editing. If the user loads their own file, an intelligent file-parser will chop up their file into the appropriate metafiles consisting of the objects detected in the file via the tagged REMARK 400 headers and the main body of the protein. These files are contained within the user’s HOME directory inside an appropriately named hidden folder and are cleaned up upon the program’s exit to conserve the space on the system. The list of parsed objects is then used to populate the associated code objects and GUI tables, complete with each of the parameters used to generate the objects. Once this initial preprocessing is done, the user is then able to manipulate each of the objects individually by either changing the size, shape, or positioning of the object in the space or by changing the atomic properties of the object such as the atomic radius, dielectric constant, atomic identifiers, or object names. The user can also add or delete individual objects and track which objects have been modified since the last compilation of the file by the color-coding of each of the object names in the list: blue for modified objects, gray for unmodified objects.

A key feature of the *ProNOI* program is the linking of the GUI controls to the molecular viewer in order to provide the user with immediate feedback. The sliders for each of the objects are linked to dynamically generated Jmol commands which construct a skeleton of the object’s expected location for the regeneration. So, even while the user is moving the sliders for the object, the object’s new position can be tracked in real-time.

Once the user’s adjustments have been made, the user can regenerate the PDB/PQR file and see exactly how the modeling configuration has changed. This operation is completed in the background by a call to the C++ object manipulation tool, which, if the appropriate executable is not found, will offer the user a helpful file navigation dialog to let them specify exactly where the program is located. The Java GUI will then process all of the parameters from each of the objects, sanitizing and validating each parameter in order to avoid harmful scripts executing on the command line, and then calling the C++ program once for each modified object. The output from the C++ program results in a single PDB/PQR file for each object which has been prefixed with a REMARK 400 header and contained in the hidden directory. These files are then combined with the original data from the PDB file and form a new compiled file in the hidden directory and loaded into the molecular viewer. These actions also preserve the user’s current perspective in the protein space which can be very useful for monitoring small changes to the objects.

The C++ object manipulation tool, which has been explained in a previous work [13] has several additional features worth mentioning. Atomic radii and charges can now be appended to each atom if the user selects the PQR file format for the output. The atomic radii of the object are simply entered into the GUI and passed through but the charges per atom are calculated via a density argument. The new version of the C++ program allows surface and volume density parameters to be passed into it in units of electron charge per Angstrom squared for surface charge density or per Angstrom cubed for volumetric charge density options. The charge per atom is then calculated by the following formulae:

(1a) $$q_{V}=d\left(\frac{q}{Å}\right)\cdot V\;q_{A}=d\left(\frac{q}{{Å}^{2}}\right)\cdot A$$

(1b) $$V_{\mathrm{sphere}}=\frac{4}{3}\pi r^{3}\;A_{\mathrm{sphere}}=4\pi r^{2}$$

(1c) $$\begin{array}{l} V_{\mathrm{Cylinder}}=\pi r^{2}\left|\vec{\text{dir}}\right|\;A_{\mathrm{Cylinder}}\\ \;=2\pi r^{2}+2\pi r\left|\vec{\text{dir}}\right| \end{array}$$

(1d) $$\begin{array}{l} V_{\mathrm{Cone}}=\frac{1}{3}\pi r^{2}h\;A_{\mathrm{Cone}}\\ \;=\pi r\left(r+\sqrt{r^{2}+{\left|\vec{\text{dir}}\right|}^{2}}\right) \end{array}$$

(1e) $$\begin{array}{l} V_{\mathrm{Box}}=\left|\vec{a}\times \vec{b}\times \vec{c}\right|\;A_{\mathrm{Box}}\\ \;=2\left[\left|\vec{a}\times \vec{b}\right|+\left|\vec{a}\times \vec{c}\right|+\left|\vec{b}\times \vec{c}\right|\right] \end{array}$$

where *q*_*v*_ and *q*_*A*_ are the charges of each atom, *d* is the given charge density with its units in parentheses as the charge of the electron per Angstroms squared or cubed, *r* is the radius of the object from the input, $\vec{dir}$ is the direction vector also from the input, $\vec{a}$ and $\vec{b}$ and $\vec{c}$ are the input vectors for the box, and *V* is the volume and A is the area of the specified object. This is then appended to each atom of the object along with the given radius in conformance to the PQR file format.

In addition to the object manipulation tools, a force field parameters selector has been added to allow the user to convert their original macromolecule PDB data into PQR format in conformance with a set of parameter files. The current force field parameters used by this program are Amber [49] (v. 98), Charmm [50] (v.22), OPLS [51] and PARSE [52] along with an option in the preferences to upload a properly formatted size (SIZ) and charge (CRG) file-set for a custom force field parameters. The custom force field parameter option is specifically useful for cases involving non-standard compounds, for which the charges and radii must be obtained with other programs, as for example with the antechamber [53]. This selector, upon object generation, scans the macromolecule PDB file for ATOM entries and attempts to find the residue and atom names and then find the corresponding radius and charge for the atom from the data read in from the force field parameter files. If the specific residue name is not found, the program will then try to find the atom name in the global id list and pull the charge and radius from there. If it is still unsuccessful, the program will record the error and set the atom’s radius to one and its charge to zero. Once all the entries have been processed and if any errors resulting from missing entries have been recorded, a window will appear displaying each of the missing entries letting the user know which entries were not found and need to be addressed.

Once the user decides to save their selections and the appropriate changes made, the compiled file will overwrite the original PDB file. Also, if the user attempts to close the program before saving their changes, the program will prompt the user to save.

## Results

Here we report *Protein Nano-Object Integrator (ProNOI)* which allows for generating atomic-style geometrical objects with user desired shape and dimensions. An unlimited number of objects can be created and combined with biological macromolecules in any given file that follows the Protein Data Bank (PDB) format. During object creation, users can use sliders to manipulate their shape, dimension, and position within the protein. In addition, the software offers the option to append charges to the objects by specifying a surface or volumetric charge density. The biological macromolecule’s atoms can be assigned charges and radii according to the user’s selection of one of four different force fields: Amber [50] (v. 98), Charmm [51] (v.22), OPLS [52] and PARSE [53]. If the object is charged, the biological macromolecules and the atomic-style objects are outputted as position, charge and radius (PQR) file; otherwise, the file remains in PDB format. If the user decides to assign different (not default) dielectric constants to the object and biological macromolecules, the output file is in position, charge, radius and epsilon (PQRE) format.

Three types of benchmark tests were conducted to determine the accuracy of the calculated energies and the versatility of the *Protein Nano-Object Integrator* as compared to DelPhi’s old style of object creation [54] and cases for which analytical solution for either the potential or electrostatic energy can be obtained. The cases with available analytical solution include: (1) a spherical dielectric cavity immersed in a highly dielectric medium with two separated charges located within the sphere [12,55]; (2) a wide cylinder representing a semi-infinite dielectric low dielectric plane and a spherical charge first positioned outside and then inside the semi-infinite plane moving along a line perpendicular to the plane surface [12,54]; (3) a charged sphere; (4) a line of charge; and (5) a charged disk. The cases for which analytical solution is not available were made by placing a protein, the bovine α-chymotrypsin-eglin C, on the four different types of objects the *ProNOI* can create and then calculating the corresponding solvation energies and comparing with the results of old DelPhi-style objects [54]. At the end, we illustrate the *ProNOI* capabilities by creating a large composite object in a shape of a robot, the Clemson Robot, which holds in its hand barnase-barstar complex.

It should be mentioned that when conducting the tests, care was taken to make sure that the parameters of the objects created by the *Protein Nano-Object Integrator* and DelPhi’s original object creation tool were as similar as possible. When creating objects with the Protein Nano-Object Integrator, the size of the atoms making up the object must be taken into consideration. For instance, when creating a sphere with a radius of 10 Å and atoms with 1 Å in radius, the object must include an offset of 1 Å in order to account for the additional length produced by the radius of the individual atoms that make up the object; otherwise, the sphere would have an effective radius of 11 Å.

The *Protein Nano-Object Integrator* has the option of varying the spacing of the atoms in the created objects. As the spacing approaches zero, there is a truer representation of a continuous dielectric medium, but this, in turn, creates larger file sizes due to the increasing amount of atoms, which increases the computational time and may cause problems visualizing the object(s) with standard molecular graphic software.

### Spherical cavity immersed in high dielectric medium

The problem of calculating the energies of two charges contained in a spherical cavity immersed in water was previously described [12,55]. The *Protein Nano-Object Integrator* was used to create a spherical object with a radius of 10 Å and an origin positioned at (0, 0, 0). An internal dielectric constant of 2 was used for the sphere and an external dielectric constant of 80, that of water, for outside of the sphere. Two charged atoms were placed inside the sphere at positions (5, 5, 0) and (5, -5, 0). Each atom carried a columbic charge of 10e. These parameters precisely follow the old-style Example 2 in DelPhi distribution (http://compbio.clemson.edu/delphi.php) but with the object creation replaced by the *Protein Nano-Object Integrator*. DelPhi was then used to calculate the total self-energy of the spherical object protein with two charges. The analytical solution to this problem is −5083.19kT.

**Figure 1:**
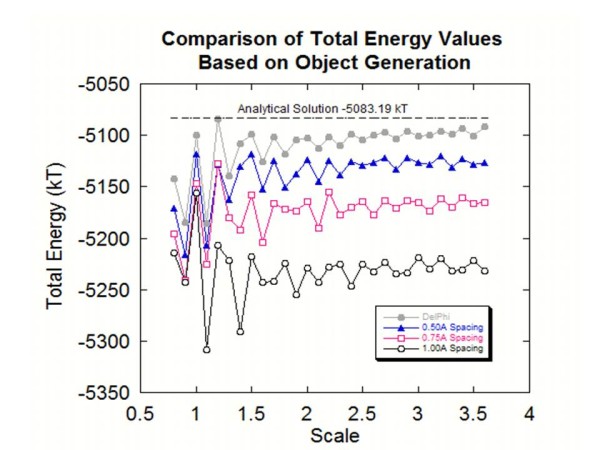
**This figure shoes the total energy values obtained from both styles of object creation with a line that represents the analytical solution for the spherical cavity, as the scale values for DelPhi are increased.** Atomic spacings of 0.50 Å, 0.75 Å and 1.00 Å were used when creating the object with the *Protein Nano-Object Integrator.*

The dielectric cavity in this case is modeled as multitude of pseudo atoms and strictly speaking is not a homogeneous cavity. However, as one decreases the spacing between pseudo-atoms forming the cavity, the model should become more homogeneous and at the limit of zero spacing should be perfect homogenous cavity. To test such an expectation, three separate spheres were created by the program with three different atomic spacings: 1.0 Å, 0.75 Å, and 0.50 Å. Figure 1 shows the calculated electrostatic energies with both the old-style DelPhi and with the object created with *ProNOI* as a function of the scale. As it was expected at large spacing between pseudo atoms forming the dielectric cavity, the calculations with *ProNOI* created object are less accurate than the old style DelPhi calculations. However, as the spacing decreases the calculated energies with *ProNOI* generated object approach the results of old-style calculations and at scale larger than 2.5grid/Å are very close to the analytical solution.

### Atom moves through semi-infinite dielectric region

The results of the solvation energy of a spherical charge approaching a semi-infinite dielectric region [12,54] (Example 3 in the DelPhi distribution) modeled by a cylinder were compared with the old-style object generation and objects generated from the *Protein Nano-Object Integrator*. The *Protein Nano-Object Integrator* was used to generate a cylinder with the exact same properties as the cylinder generated in the old-style Example 3. The coordinates of the cylinder were A (0, 25, 0), B (0,-25, 0) and a radius of 30 Å. The internal dielectric constant of the sphere was 2.0 and the external dielectric was 80.0, that of water. A probe radius of 0.2 Å was used in this test.

**Figure 2:**
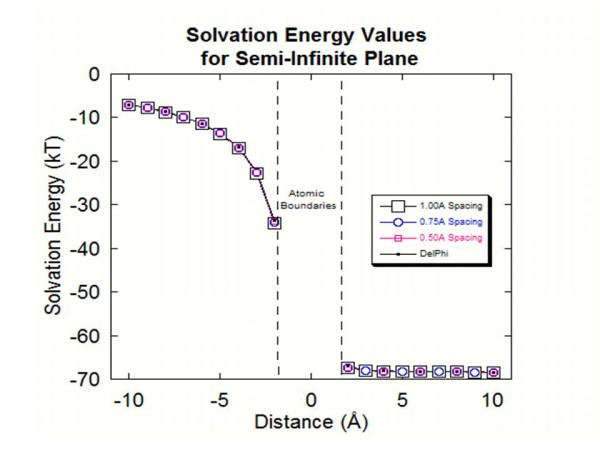
**This figure shows the solvation energy values obtained from both styles of object creation for the semi-infinite plane test.** The graph shows the values for four different objects using 0.50 Å, 0.75 Å and 1.00 Å atomic spacings with the *Protein Nano-Object Integrator* and a fourth object created using DelPhi’s object creation. In the center of the graph there are two dotted lines that represent the surface boundaries of the semi-infinite plane for which an analytical solution does not exist.

A charged sphere with a dielectric constant of 2.0, charge of 1.0e, and radius of 1.0 Å was initially placed inside of the cylinder 10 Å from the surface boundary of the cylinder. The charged sphere was then moved 1 Å stepwise toward, and then outside of the boundary of the cylinder (to a max distance of 10 Å outside of the cylinder). At each step the solvation energies were compared between the old and new-style object generation. As done in the previous example, atomic spacings of 1.0 Å, .75 Å, and .50 Å were used to demonstrate how the increased density of generated atoms leads to more accurate results. Figure 2 shows the solvation energy values obtained for each of the atomic spacings and for comparison the same done with the old-style DelPhi created object. An analytical solution, for positions that the probe sphere does not touch the interface, was obtained by using the method of images (see example [56] for more details). As can be seen, the all the values are very close to the analytical solution, especially for probe sphere positions away from the interface.

### Charged sphere

**Figure 3:**
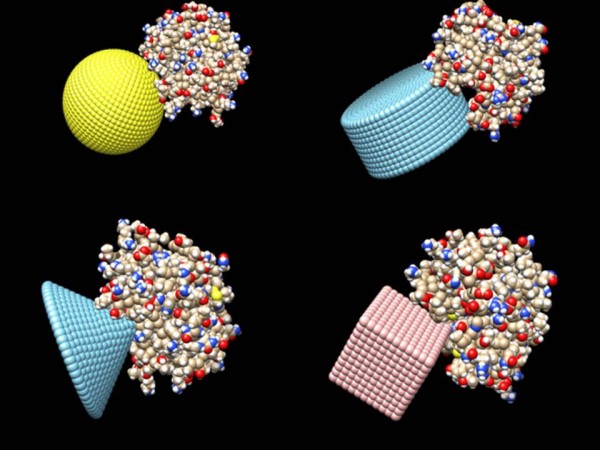
This figure was created in Chimera in order to give a visual representation of the protein 1ACB attached to the four test objects.

A spherical object was generated by the *ProNOI* with a radius of 2.0 Å and an atomic spacing of 1.0 Å. A charge of one electron unit was given to each of the atoms that make up the sphere. The analytical solution was obtained via Coulomb law of a homogeneously charged sphere. The potential was calculated with DelPhi at distances of 1 Å to 40 Å from the sphere. The DelPhi calculations were then compared to analytical solutions for this problem (Figure 3). The distances from 1 Å to 4 Å were omitted in the graph due to the sphere’s size making the point charge modeling erroneous for these short distances from the sphere’s surface. However, as the distance increases toward infinity (Figure 3) the results steadily approach the analytical solution.

### Line of charge

**Figure 4:**
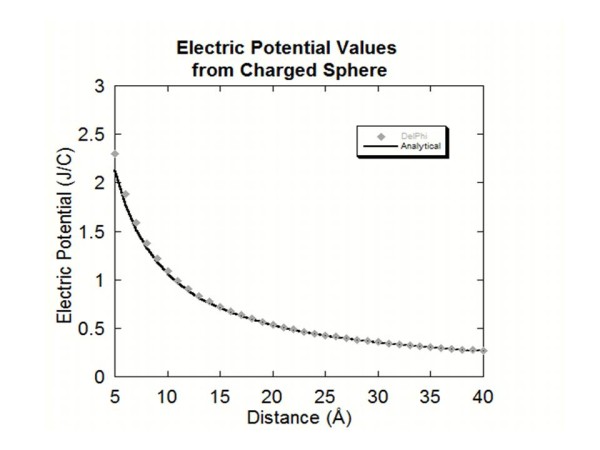
This figure shows the electric potential values obtained for each distance away from the charged sphere along with the analytical solution.

A line of charge was generated using the *ProNOI* by creating a cylinder of radius 0.50 Å, height of 40 Å, atomic spacing of 1.0 Å and a charge of 1e to each atom making up the line. For the analytical solution, the object was then modeled as a finite line charge and the electric potential values for distances of 1 Å to 40 Å from the line charge. The same calculations were done with DelPhi as well. The DelPhi calculations were then compared to analytical solutions for this problem (Figure 4). The distances from 1 Å to 4 Å were omitted in the graph due to the individual atoms size making the point charge modeling erroneous for these short distances from the line of charge. However, as the distance increases toward infinity (Figure 4) the DelPhi results are practically identical with analytical solution.

### Disc of charge

**Figure 5:**
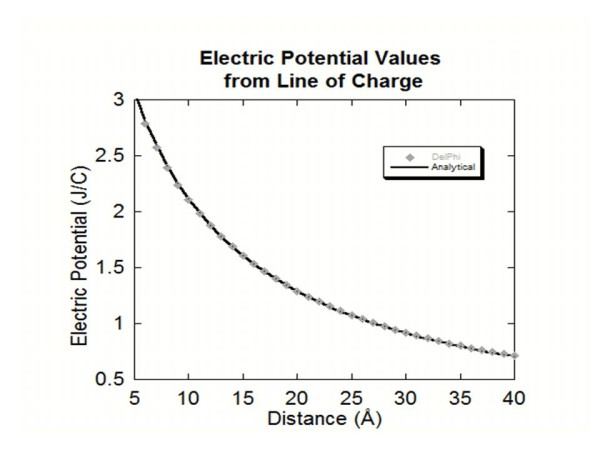
This figure shows the electric potential values obtained for each distance away from the line of charge along with the analytical solution.

A disc of charge was generated using the *ProNOI* by creating a cylinder of radius 5 Å, width of 0.50 Å, atomic spacing of 1.0 Å and a charge of 1e to each atom making up the disc. The object was then modeled as a thin disc of charge and the electric potential values for distances of 1 Å to 40 Å from the disc of charge were calculated analytical formula. Same calculations were done with DelPhi as well. The DelPhi calculations were then compared to analytical solutions for this problem (Figure 5). The distances from 1 Å to 4 Å were omitted in the graph due to the individual atoms size making the disc of charge modeling erroneous for these short distances from the line of charge. However, as the distance increases toward infinity (Figure 5) the DelPhi results are identical with analytical solution.

### Protein placed on created objects

**Figure 6:**
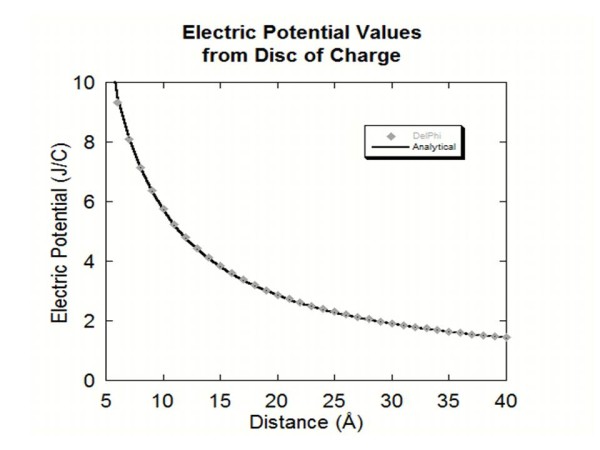
This figure shows the electric potential values obtained for each distance away from the disc of charge along with the analytical solution.

Four separate objects were created by the *Protein Nano-Object Integrator*: sphere, cylinder, cone and cube. The protein complex, the bovine α-chymotrypsin-eglin C, PDB ID 1ACB, was placed onto these objects, just touching the surface. The objects were created to have a comparable size to the length of the protein while still keeping the file sizes of the objects at a manageable size (40 Å was used). The sphere had a radius of 20 Å. The cylinder had a radius of 20 Å and a height of 40 Å. The cone had a base diameter of 40 Å and an opening angle of 45 degrees. The cube had a length, width and height of 20 Å in order for the diagonal of the cube to share a comparable length to the test protein. Depending upon the case, either the object or protein was rotated to make sure of the best fit of the protein onto the surface of the object, while still making sure that the protein did not cross the external boundary of the object. Figure 6 shows the configuration at which the proteins were placed onto the objects.

Solvation energies were calculated by DelPhi with a probe radius of 1.4 and atomic spacing of 2.0 Å for each of the four examples and compared to the old-style object creation for identical sized objects. As can be seen from Table 1, the percent differences between the two styles are very small. Even with a relatively large atomic spacing of 2.0 Å as compared to the previous examples, the percentages are very low.

**Table 1:** **This table shows the solvation energies calculated by DelPhi by placing the protein 1ACB on four objects each created by the *Protein Nano-Object Integrator***

	**DelPhi (kT)**	**PNO (kT)**	**% Difference**
**Sphere**	−27955.27626	−27960.97555	0.02
**Cylinder**	−27960.22117	−27948.76093	0.04
**Cone**	−27973.12424	−27959.91403	0.05
**Cube**	−27957.72562	−27959.3489	0.01

The final column shows the percent difference value between the two object creation styles respective to each object type.

### Clemson robot

To illustrate the capabilities of the ProNOI, we created a composite object in a shape of a robot and called it the Clemson Robot. The dimensions of the figure are 312 × 729 × 292 Angstroms. The Clemson Robot holds in its hand the barstar-barnase complex, PDB ID 1BRS. To further illustrate the option of charging the objects, we equipped the Clemson Robot with volumetric charges. The charge distributions are: head: +2.0 e, body: -4.0 e, each arm: +1.0 e, each leg: +1.0 e, each foot: -1.0 e. This distribution makes the total net charge of the robot to be 0. In each part, the charge density is a constant according to the total charges and atom numbers.

**Figure 7:**
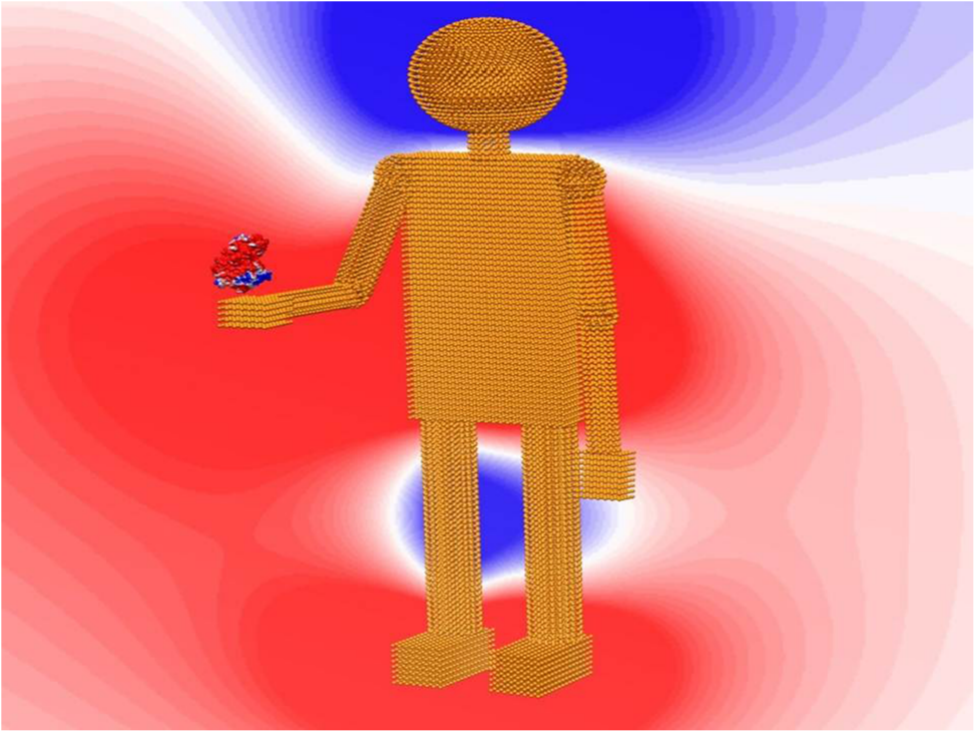
This figure is generated by Chimera, the parameters used in Chimera are set as below: color of protein surface: from −2.0(red) to 2.0(blue) color of background contour map: from −1.0(red) to 0.6 (blue).

The calculations of the potential and solvation energy were done with parallelized DelPhi [11]. The main parameters we set in DelPhi are scale = 1, perfil = 70, the resulting grid size is 1041*1041*1041. The dielectric constant in the protein and robot is set as 2.0, which in the water is set as 80.0. The reaction field energy from DelPhi calculation is −1872.78 kt. The potential distribution and the corresponding structure of the Clemson Robot are shown in Figure 7.

The Clemson Robot PDB and PQR files are available for download from http://compbio.clemson.edu/delphi.php -- > Clemson Robot files.

## Conclusions

The *ProNOI* is convenient tool for generating atomic-style shapes in conjunction with biological macromolecule(s). Charges on the macromolecules atoms and the atoms making the shapes are assigned according to user preferences to allow various scenarios of modeling. Each object and macromolecule can be assigned a user selected dielectric constant. The output file is in PDB, PQR or PQRE format which is readable by almost any software available in biophysical field.

## Availability and requirements

Project name: *Protein – Nano Object Integrator (ProNOI)*

Project home page: http://compbio.clemson.edu/downloadDir/ProNO_integrator.tar.gz

Operating system(s): Linux OS or CentOS

Programming language: Java and C++

Other requirements: e.g. Java 1.3.1 or higher, Tomcat 4.0 or higher.

License: None.

Any restrictions to use by non-academics: None.

## Abbreviations

ProNOI: Protein Nano-Object Integrator; PBE: Poisson boltzmann equation; MD: Molecular dynamics; PQR format: Position, charge and radius format; PQRE format: Position, charge, radius and epsilon format.

## Competing interests

The authors declare that they have no competing interests.

## Authors' contributions

NS: developed the ProNOI algorithm, wrote the code and integrated it with Jmol; BC: designed the test cases, including “Clemson Robot” and performed the benchmarking; LL: carried benchmarking on “Clemson Robot”, CL: carried benchmarking on “Clemson Robot”; EA: designed and supervised the project. All authors wrote the manuscript. All authors read and approved the final manuscript.
